# Dynamic reconfiguration of brain functional networks in world class gymnasts: a resting-state functional MRI study

**DOI:** 10.1093/braincomms/fcaf083

**Published:** 2025-02-19

**Authors:** Bolin Cao, Yu Guo, Fengguang Xia, Lunxiong Li, Zhanbing Ren, Min Lu, Jun Wang, Ruiwang Huang

**Affiliations:** School of Psychology, Center for Studies of Psychological Application, Guangdong Key Laboratory of Mental Health and Cognitive Science, Key Laboratory of Brain, Cognition and Education Sciences, Ministry of Education, South China Normal University, Guangzhou 510631, China; School of Psychology, Center for Studies of Psychological Application, Guangdong Key Laboratory of Mental Health and Cognitive Science, Key Laboratory of Brain, Cognition and Education Sciences, Ministry of Education, South China Normal University, Guangzhou 510631, China; Institute for Brain Research and Rehabilitation, South China Normal University, Guangzhou 510631, China; Key Laboratory of Brain, Cognition and Education Science, Ministry of Education, China; Institute for Brain Research and Rehabilitation, and Guangdong Key Laboratory of Mental Health and Cognitive Science, South China Normal University, Guangzhou 510631, China; Department of Physical Education, Shenzhen University, Shenzhen 518060, China; Institute of Psychology, Chinese Academy of Sciences (Key Laboratory of Mental Health, Chinese Academy of Sciences), Department of Psychology, University of Chinese Academy of Sciences, Beijing 100101, China; Faculty of Psychology, State Key Laboratory of Cognitive Neuroscience and Learning and IDG/McGovern Institute for Brain Research, Beijing Normal University, Beijing 100875, China; School of Psychology, Center for Studies of Psychological Application, Guangdong Key Laboratory of Mental Health and Cognitive Science, Key Laboratory of Brain, Cognition and Education Sciences, Ministry of Education, South China Normal University, Guangzhou 510631, China

**Keywords:** modularity, motor skill learning, multilayer network, neuroplasticity, temporal core-periphery organization

## Abstract

Long-term intensive training has enabled world class gymnasts to attain exceptional skill levels, inducing notable neuroplastic changes in their brains. Previous studies have identified optimized brain modularity related to long-term intensive training based on resting-state functional MRI, which is associated with higher efficiency in motor and cognitive functions. However, most studies assumed that functional topological networks remain static during the scans, neglecting the inherent dynamic changes over time. This study applied a multilayer network model to identify the effect of long-term intensive training on dynamic functional network properties in gymnasts. The imaging data were collected from 13 gymnasts and 14 age- and gender-matched non-athlete controls. We first construct dynamic functional connectivity matrices for each subject to capture the temporal information underlying these brain signals. Then, we applied a multilayer community detection approach to analyse how brain regions form modules and how this modularity changes over time. Graph theoretical parameters, including flexibility, promiscuity, cohesion and disjointedness, were estimated to characterize the dynamic properties of functional networks across global, network, and nodal levels in the gymnasts. The gymnasts showed significantly lower flexibility, cohesion and disjointedness at the global level than the controls. Then, we observed lower flexibility and cohesion in the auditory, dorsal attention, sensorimotor, subcortical, cingulo-opercular and default mode networks in the gymnasts than in the controls. Furthermore, these gymnasts showed decreased flexibility and cohesion in several regions associated with motor function. Together, we found brain functional neuroplasticity related to long-term intensive training, primarily characterized by decreased flexibility of brain dynamics in the gymnasts, which provided new insights into brain reorganization in motor skill learning.

## Teaser

Cao *et al*. report the properties of network dynamic reconfiguration in world class gymnasts by using a multilayer network analysis and found decreased flexibility across multiple scales compared with non-athlete controls. This result suggests that long-term intensive training may promote a more stable and efficient brain functional configuration.

## Graphical Abstract

**Graphical Abstract:**
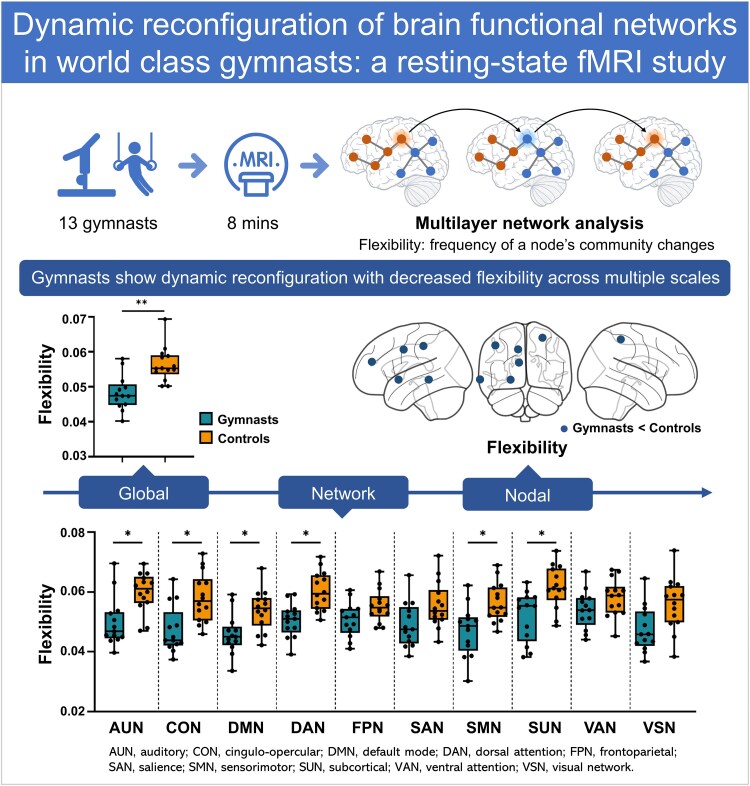

## Introduction

World class gymnasts (WCGs) represent the peak of motor skills, showing outstanding balance, strength and flexibility. Their exceptional performances are acquired through long-term intensive training, often initiated in early childhood and sustained throughout their athletic careers.^1^ Their excellent athletic performances capture public attention and raise the question of whether their brain function and structure differ from those of non-athletes.^2^

The Brian network approach^3,4^ has been widely used to study the brain functional mechanism underlying athletic performance.^1,5-7^ For example, Wang *et al*.^1^ investigated the topological properties of brain’s functional networks in these gymnasts by using the resting-state fMRI (rs-fMRI) data. Their study identified significant functional reorganizations within the cerebellum, frontoparietal (FPN) and cingulo-opercular network (CON) in WCGs compared with controls. Subsequently, Huang *et al*.^5^ observed that long-term intensive training could optimize both intra- and inter-network functional connectivity (FC) related to motor and executive functions in WCGs. These studies provided functional evidence that training-associated neuroplasticity is related to brain modular organization of brain connectivity.^8-10^ Modularity is an important topological property for functional brain networks and refers to the formation of distinct network communities within the brain that are densely connected internally but sparsely connected with other such communities.^8,9,11^ The brain’s modular architecture could facilitate efficient information processing^12^ and conservation of neural resources, promoting rapid adaptability to environmental changes^13^ and maintaining functional specialization within networks.^10^

However, these rs-fMRI studies assumed that the resting-state FC is static, overlooking the inherently dynamic nature of the brain.^1,5^ Dynamic FC (dFC) analysis provides a framework to capture temporal fluctuations in functional connectivity patterns, enabling us to examine how brain activity adapts to different cognitive tasks and demands.^14-16^ Recently, some studies applied this method to investigate the influence of long-term intensive training on brain function.^17-19^ For example, several studies acquired the rs-fMRI data from table tennis players and identified the dynamic functional changes in brain regions related to motor and visual networks due to motor training.^18,19^

This study applied a multilayer network approach^20^ to analyze the temporal transitions and modular reconfigurations within brain functional networks for the WCGs. This approach has been widely used to reveal how brain regions form modules and how these configurations shift over time in rs-fMRI data.^9,21-23^ Flexibility, a key statistical parameter of network reconfiguration, reflects how often a brain region changes its modular affiliation. Several lines of evidence have shown that the dynamic reconfiguration of functional modular structures, especially flexibility, is closely associated with developing motor skills,^9,17,21,24^ musical skills acquiring,^25^ and individual differences in higher-order cognition.^26^ Gymnastics, which demands rapid and efficient coordination among sensory, motor and high-order networks to perform complex actions, offers a unique model to study how the brain dynamically reorganizes its modular structure in response to intensive long-term training.

This study hypothesized that long-term intensive training may lead to distinct patterns of dynamic functional network reorganization in the WCGs across multiple scales, including the whole brain, network and nodal levels. To this end, we collected the rs-fMRI data from 13 WCGs and 14 age and gender-matched non-athlete controls. We constructed dFC matrices for each subject using a sliding window approach. Multilayer community detection was then applied to these dFC matrices to identify community assignments of brain regions. Finally, several dynamic network parameters, including flexibility, promiscuity, cohesion and disjointedness, were calculated across multiscale to characterize dynamic functional network properties.

## Materials and methods

### Subjects

This study analyzed the rs-fMRI data obtained from thirteen world class gymnasts who won at least one gold medal in the Gymnastic World Cup or Olympic Games (7F/6M, aged 17–26 years, mean ± SD 20.5 ± 3.2 years). The detailed demographic information for each gymnast is presented in Table 1. These gymnasts started training at an average age of 4.5, with a mean training time of 6 h/day. By the time of this scan, they had amassed an average of over 12.5 years of training. Furthermore, fourteen non-athlete volunteers of comparable age and gender (7F/7 M, 19–28 years old, mean ± SD = 20.5 ± 2.5 years old) with no gymnastics training were enlisted as the control group. There was no significant difference in age (*t* = −1.33 and *P* = 0.20) and gender (*χ^2^* = 0.04, *P* = 0.84) between the WCGs and controls. None of the subjects had a record of psychiatric or neurological disorders, movement disorders, or brain injuries. The Institutional Review Board of the State Key Laboratory of Cognitive Neuroscience and Learning at Beijing Normal University approved the study. Written informed consent was obtained from each subject prior to the experiment.

**Table 1:** Characteristics of the world class gymnasts (WCGs)

No.	Best medal record since 2007	Gender	Age (yrs)	Age of commencement (yrs)	Years of training
01	OC	M	24	4.5	19.5
02	WC	M	24	4.5	19.5
03	WC	M	23	4.5	18.5
04	WC	M	26	4.5	21.5
05	WC	M	21	4.5	16.5
06	WC	M	23	4.5	18.5
07	WC	F	19	4.5	14.5
08	OC	F	18	3.5	14.5
09	OC	F	21	4.5	16.5
10	WC	F	17	4.5	12.5
11	OC	F	17	4.5	12.5
12	OC	F	17	4.5	12.5
13	OC	F	17	4.5	12.5

All gymnasts had won individual or team gold medals in the Gymnastics World Cup or Olympic Games between 2007 and 2010. OC, Olympic Champions; WC, World Champions, or World Cup Champions; M/F, male/female; yrs, years old.

### MRI data acquisition

The MRI data were obtained on a 3T Siemens Trio Tim MR scanner with a 12-channel phased-array head coil at the Brain Imaging Center of Beijing Normal University. The rs-fMRI data were acquired using a gradient-echo EPI sequence with the following parameters: repetition time (TR) = 2000 ms, echo time = 30 ms, flip angle = 90^°^, field of view = 224 mm × 224 mm, data matrix = 64 × 64, slice thickness/gap = 3.6 mm/0.7 mm, 33 interleaved transversal slices and 240 volumes acquired in about 8 min. In addition, high-resolution brain structural images were obtained using a T1-weighted 3D magnetization-prepared rapid gradient-echo sequence. The sequence parameters were as follows: TR/echo time = 1900ms/3.44 ms, slice thickness = 1 mm, ﬂip angle = 9^°^, field of view = 256 mm × 256 mm, data matrix = 256 × 256 and 176 sagittal slices. During the rs-fMRI scan, the subjects were asked to keep their eyes closed but not think about particular things. Both the functional and structural images were obtained for each subject in the same session.

### Data preprocessing

The rs-fMRI data were preprocessed using DPABI^27^ and SPM12 (http://www.fil.ion.ucl.ac.uk/spm/software/spm12). We removed the first 10 volumes and performed slice timing and head motion corrections. We co-registered the functional images to the individual structural images and normalized them in the MNI standard space. The nuisance variables were regressed, including 24 head movement parameters^28^ from head motion correction and the mean signals of brain white matter, cerebrospinal fluid and linear trend. Finally, the functional images were band-pass filtered using 0.01–0.1 Hz and were smoothed with a Gaussian kernel of 6 mm full width at half maximum. The mean framewise displacement^29^ was computed for each subject and compared between the groups (WCG: 0.14 ± 0.07, controls: 0.12 ± 0.05, *t* = −1.33 and *P* = 0.20). The inclusion criterion of head movement for subjects was set as mean framewise displacement < 0.3, and none were excluded.

### Multilayer network construction

A multilayer network consists of the following components: (i) nodes representing different brain regions, (ii) layers representing various time windows, (iii) state nodes, where each state node corresponds to a specific brain region within a particular layer and (iv) weighted edges that connect state nodes, reflecting the interactions between brain regions across different layers.^30^

This study used the Power-264 atlas^31^ to define the nodes. We first removed 37 regions of interest (ROIs) that did not align with well-established networks based on previous studies.^32-34^ The remaining 227 ROIs, comprising 214 cortical and 13 subcortical regions, were assigned to ten resting-state functional networks^31^ as follows: sensorimotor network (SMN, 35 ROIs), cingulo-opercular network (CON, 14 ROIs), auditory network (AUN, 13 ROIs), default mode network (DMN, 58 ROIs), visual network (VSN, 31 ROIs), frontoparietal network (FPN, 25 ROIs), salience network (SAN, 18 ROIs), subcortical network (SUN, 13 ROIs), ventral attention network (9 ROIs) and dorsal attention network (DAN, 11 ROIs). We extracted the mean time series from the 227 ROIs with a 5 mm diameter sphere.

**Figure 1:**
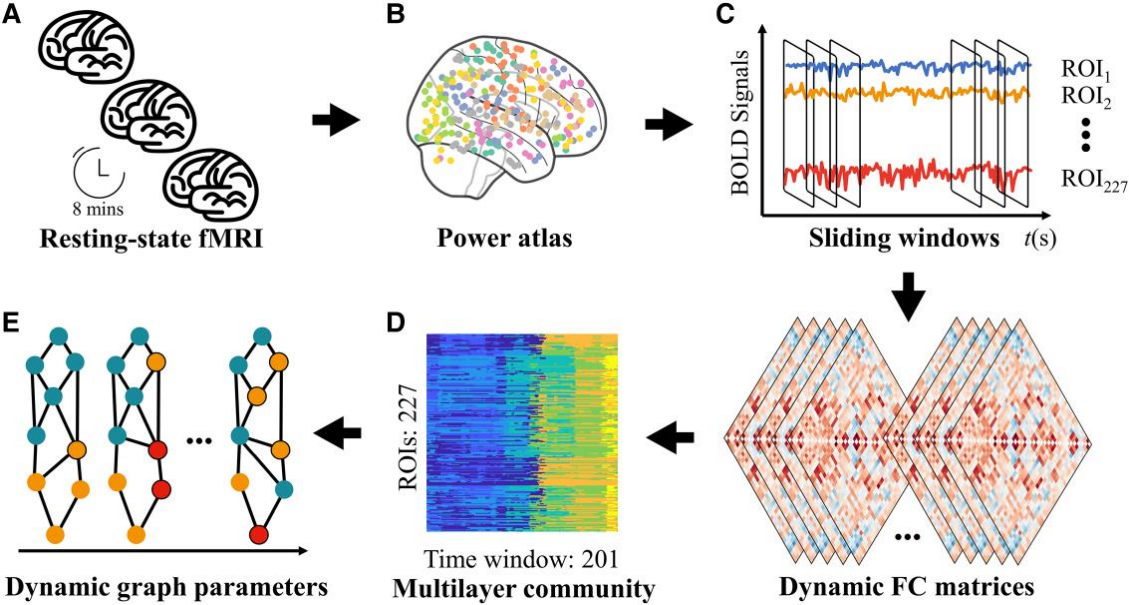
**Flowchart of the multilayer network analysis.** (**A)** This study recruited 13 WCGs and 14 age- and gender-matched non-athlete controls. The resting-state fMRI data were acquired in 8 min for each subject. (**B)** After preprocessing the functional data, we extracted the mean time series from the selected 227 ROIs using Power Atlas. (**C)** We applied the sliding window approach to construct the dFC with a window width = 30 TRs and step = 1 TR. (**D)** Using the Louvain community detection algorithm, we estimated the multilayer community based on the dFC matrices. (**E)** The dynamic network parameters, including flexibility, promiscuity, cohesion and disjointedness, were calculated, and the differences between the two groups were compared. dFC, dynamic functional connectivity; WCGs, world class gymnasts.

The sliding window approach was used to decompose the time series (230 TRs) into smaller, overlapping segments for each subject (Fig. 1). Previous rs-fMRI studies^14,35^ have suggested that 30–60 s time windows are reasonable in constructing dynamic functional connectivity because this duration balances the need for capturing meaningful temporal variations in brain activity while maintaining sufficient statistical power. Thus, we set the window length = 30 TRs and the sliding step = 1 TR, generating 201 windows or layers. For each subject, we estimated time-resolved dFC, yielding a matrix of size *N* × *N* × *L*, where *N* = 227 is the number of brain regions, and *L* = 201 is the total number of time points. This process produced an undirected, weighted connectivity matrix to detect community architecture for each subject.

Then, we estimated the brain community for each subject using an iterative Louvain multilayer modularity algorithm.^20^ Brieﬂy, this algorithm partitions the communities in a multilayer network by optimizing the multilayer modularity quality function *Q* as follows:


$$\begin{array}{c} (Q,\;S)=\frac{1}{2\mu }\underset{ijlr}{\sum }\;[(A_{ijl}-\gamma_{l}P_{ijl})\delta_{lr}+\delta_{ij}\omega_{\;jlr}]\delta (g_{il},g_{\;jr}) \end{array}$$

where *μ* is the sum of edge weights across all nodes and layers. $A_{ijl}$ is the FC value between nodes *i* and *j* at layer *l*, while $P_{ijl}$ represents the corresponding null model matrix given by the Newman-Girvan null model. The parameter $\gamma_{l}$ is the topological resolution of layer *l*, related to the edge weight of the intralayer network, while $\omega_{jlr}$ is the connection strength between node *i* in layer *l* and layer *r*. The temporal coupling parameter *ω* sets the edge weight of the interlayer network. This study set *γ* and *ω* to default (*γ* = *ω* = 1) by following previous studies.^22,36,37^ The parameter $g_{il}$ and $g_{\;jr}$ represents the community assignments for nodes *i* and *j* across the temporal layers *l* and *r*, respectively. The Kronecker’s *δ*-function $\delta (g_{il},g_{\;jr})$ equals to 1 if $g_{il}$ = $g_{\;jr}$, otherwise 0. The *S* matrix provides the community assignments for each node across all layers, with dimensions of 227 × 201.

### Dynamic network parameters

This study applied flexibility, promiscuity, cohesion and disjointness to characterize the dynamics of multilayer networks for each subject. Flexibility describes the number of times a node changed communities, normalized by the number of possible changes.^9^ The flexibility of node *i* is defined as:


$$\begin{array}{c} F_{i}=\frac{m}{L-1} \end{array}$$

where *L* is the number of windows or multilayers *L* = 201, and *m* is the number of times the node *i* changes its community.^38^ The flexibility value ranges from 0 (no change across the community) to 1 (change of community in each layer). Regions with high ﬂexibility are thought to have a larger tendency to interact with diﬀerent networks.

Additionally, flexibility has been used to identify the temporal core-periphery organization in brain networks.^39^ This temporal core-periphery organization is a fundamental property of brain network architecture that distinguishes between less flexible nodes, which are consistently affiliated with their own community (temporal core), and more flexible nodes, which frequently change their affiliation to different communities (temporal periphery).^22^ To explore the effect of long-term intensive training on this organization, this study set nodes with the lowest 10% flexibility as the temporal core and those with the highest 10% as the temporal periphery for the WCGs and controls.

Promiscuity is defined as the functional diversity of a node by quantifying the fraction of all possible communities a node engages with over time.^40^ For node *i*, its promiscuity is given as follows:


$$\begin{array}{c} P_{i}=\frac{c_{p}}{C} \end{array}$$

where *c_p_* is the community in which the node is involved in, and *C* is the total community. A high promiscuity (maximum is 1) represents a node that participates in each community at least once. A low promiscuity (minimum is 1/*N*) means a node consistently remains in the same community.

We also estimated cohesion and disjointedness^21^ to measure how groups of nodes change communities. Although flexibility and promiscuity provide information on the number of community changes and the number of communities visited by a node, they cannot indicate whether these changes occur collectively or independently.^21^ Cohesion is the extent to which nodes change communities together, capturing the number of times a node changes its community allegiance in coordination with at least one other node from its previous community.^30^ High cohesion values indicate that nodes frequently change communities together with other nodes, whereas low cohesion values suggest that nodes change communities less often in coordination with other nodes. In contrast, disjointedness measures the frequency with which a node changes communities independently without being accompanied by other nodes from its previous community.^30^

### Statistical analyses

Because of the inherent randomness of the Louvain community detection algorithm, we repeated this algorithm 100 times. For each repetition, we calculated flexibility, promiscuity, cohesion and disjointness and then averaged the value of these parameters to obtain the final estimated values.

A two-sample *t*-test was used to test between-group differences in flexibility, promiscuity, cohesion and disjointedness across the nodal-, network- (averaging nodes within a brain network) and global level (averaging across all 227 node values). We generated an empirical distribution for a given parameter by randomly redistributing all subjects’ parameter values into two groups and recalculating the *t*-value based on 5000 permutation tests. A significance level of *P* < 0.05 was set to determine significant differences, using the false discovery rate (FDR) correction to control multiple comparisons. In the calculations, we took age, gender and mean framewise displacement as covariates.

### Robustness analysis

Due to the ongoing debate regarding optimal parameters in the sliding window approach,^41^ we conducted an additional analysis to evaluate the impact of different sliding window parameters on the robustness of our results from the WCGs. Specifically, we varied the width of the time windows and the step as follows: (i) window width = 30 TRs and sliding step = 2 TRs, (ii) window width = 40 TRs and sliding step = 1 TR and (iii) window width = 20 TRs and sliding step = 1 TR. We also assessed how variations in educational background influenced the study results.

## Results

### Between-group differences in dynamic network parameters

**Figure 2:**
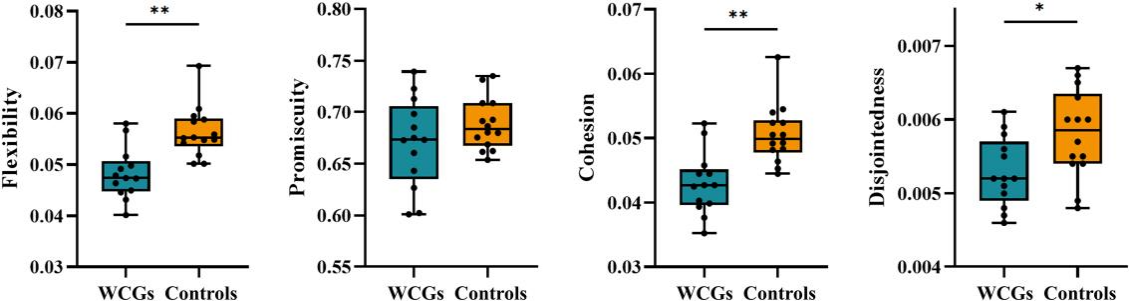
**Between-group differences in flexibility, promiscuity, cohesion and disjointedness at the global level between the WCGs and non-athlete controls.** The black points represent individual data values for dynamic graph metrics. The boxplot displays the median, with the upper and lower quartiles indicating the interquartile range. The whiskers extend to the minimum and maximum values. Refer to the ‘Result’ section for further statistical details. Asterisks (*) indicate statistical significance (*P < 0.05; **P < 0.01, two-sample *t*-test, FDR correction). WCGs, world class gymnasts.

On the global level, the WCGs had significantly lower flexibility (*t* = −3.526, *P* = 0.002), cohesion (*t* = −3.451, *P* = 0.003) and disjointedness (*t* = −2.598, *P* = 0.020) than in the controls (Fig. 2). However, no significant difference (*t* = −0.904, *P* = 0.375) in promiscuity was found between the WCGs and controls.

**Figure 3:**
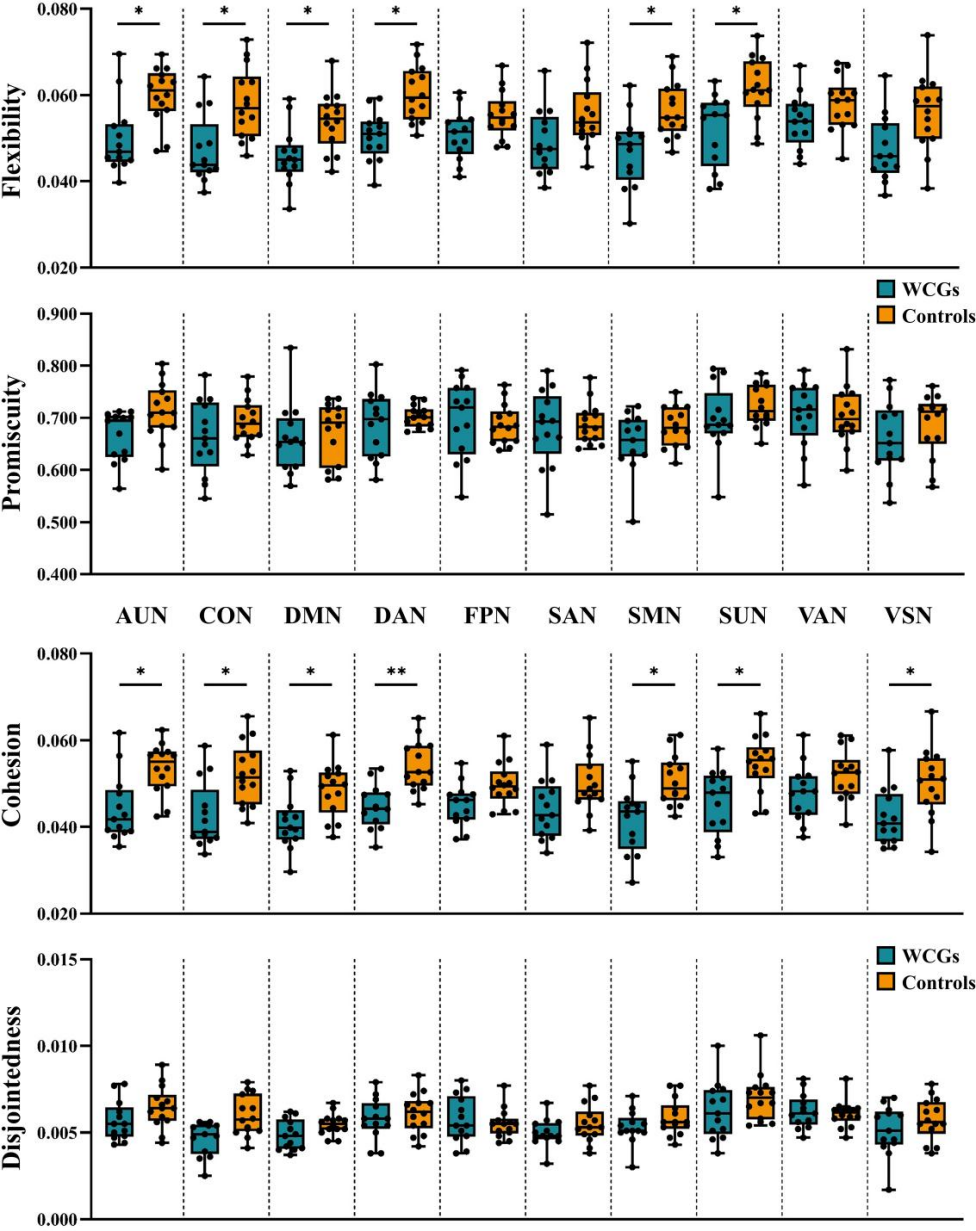
**Group differences in the dynamic network parameters (flexibility, promiscuity, cohesion and disjointedness) at the network level between the WCGs and non-athlete controls.** The black points represent individual data values for dynamic graph metrics. The boxplot displays the median, with the upper and lower quartiles indicating the interquartile range. The whiskers extend to the minimum and maximum values. Refer to Table 2 for statistical details. The asterisk * denotes that results are significant (*P < 0.05; **P < 0.01, two-sample *t*-test, FDR correction). SMN, sensorimotor network; CON, cingulo-opercular network; AUN, auditory network; DMN, default mode network; VSN, visual network; FPN, frontoparietal network; SAN, salience network; SUN, subcortical network; VAN, ventral attention network; DAN, dorsal attention network; WCGs, world class gymnasts.

Figure 3 shows between-group differences in flexibility, promiscuity, cohesion and disjointedness at the network level. The WCGs showed lower flexibility and cohesion in the resting-state networks, including the AUN, CON, DMN, DAN, SMN and SUN, than the controls. In addition, the WCGs had lower cohesion in the VSN than the controls. No significant difference in network-level promiscuity and disjointedness was found between the WCGs and controls. The detailed information for flexibility, promiscuity, cohesion and disjointedness for different groups is listed in Table 2.

**Table 2:** Significant difference in the network-level flexibility, cohesion and disjointedness between the WCGs and non-athlete controls

Network	Flexibility		Promiscuity		Cohesion		Disjointedness	
	*t-*value	*P*_(FDR)_	*t-*value	*P*_(FDR)_	*t-*value	*P*_(FDR)_	*t-*value	*P*_(FDR)_
AUN	−3.129	0.027*	−2.053	0.566	−3.090	0.027*	−1.919	0.220
CON	−2.993	0.027*	−1.073	0.818	−2.832	0.032*	−3.032	0.078
DMN	−2.437	0.046*	−0.177	0.958	−2.397	0.038*	−1.967	0.220
DAN	−3.755	0.020*	−0.926	0.818	−3.947	0.006*	−1.047	0.377
FPN	−1.690	0.126	0.317	0.958	−2.050	0.060	0.445	0.724
SAN	−1.824	0.096	0.054	0.958	−1.847	0.098	−1.261	0.377
SMN	−2.847	0.027*	−1.473	0.773	−2.896	0.027*	−1.795	0.233
SUN	−2.581	0.035*	−0.489	0.958	−2.595	0.033*	−1.103	0.377
VAN	−1.308	0.214	0.095	0.958	−1.348	0.199	0.013	0.990
VSN	−2.228	0.059	−0.844	0.818	−2.299	0.041*	−1.203	0.377

A positive (negative) *t*-value indicates that the dynamic network parameter was significantly higher (lower) in the WCGs than in the controls. SMN, sensorimotor network; CON, cingulo-opercular network; AUN, auditory network; DMN, default mode network; VSN, visual network; FPN, frontoparietal network; SAN, salience network; SUN, subcortical network; VAN, ventral attention network; DAN, dorsal attention network.

Asterisks (*) indicate statistical significance (*P < 0.05; FDR correction).

**Figure 4:**
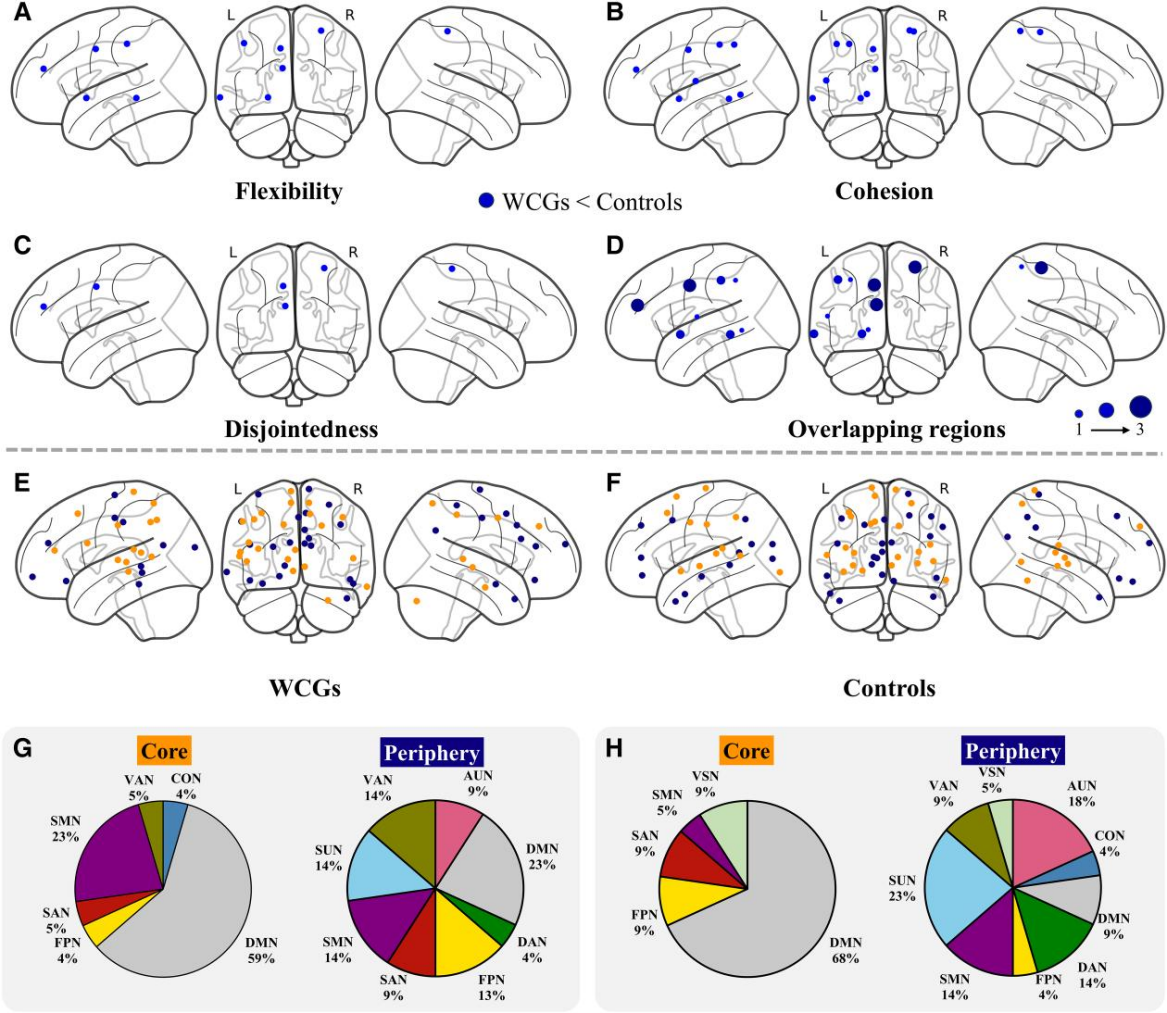
**Brain regions with significant group differences in the dynamic network properties of brain functional networks at the nodal level.** The nodes showed between-group differences in (**A**) flexibility, (**B**) cohesion and (**C**) disjointedness between the world class gymnasts (WCGs) and non-athlete controls. The blue dots represented the brain regions where the WCGs showed lower values than in the controls. Two-sample *t*-tests were used for statistical analyses, with detailed results provided in Table 3. (**D**) The overlapping ROIs with between-group differences in flexibility, cohesion and disjointedness. Dot size represented the number of measures with group differences: small for one, medium for two, and large for all three. Table 3 lists the detailed information on these ROIs. Brain plots showed the distribution of temporal core nodes (top 10%, represented by orange dots) and temporal periphery nodes (bottom 10%, represented by blue dots) in the (**E**) WCGs and (**F**) controls. The pie charts further showed the network proportion of these temporal core and periphery nodes in the (**G**) WCGs and (**H**) controls. SMN, sensorimotor network; CON, cingulo-opercular network; AUN, auditory network; DMN, default mode network; VSN, visual network; FPN, frontoparietal network; SAN, salience network; SUN, subcortical network; VAN, ventral attention network; DAN, dorsal attention network; WCGs, world class gymnasts.

Figure 4A–C shows the nodallevel between-group differences of these dynamic parameters. Specifically, the WCGs showed significantly lower flexibility in six regions, including the right postcentral gyrus (PostCG), left middle cingulate gyrus (MCG), left middle temporal gyrus (MTG), left angular gyrus and left putamen than the controls (Fig. 4A). These gymnasts also exhibited decreased cohesion in 10 regions, including the right PostCG, left MCG, left Rolandic operculum, left MTG, left angular gyrus, left lingual gyrus, left putamen, left superior parietal gyrus and left inferior parietal gyrus compared with the controls (Fig. 4B). In addition, the gymnasts also showed lower disjointedness than the controls in three nodes, the right PostCG, left MCG and left MTG (Fig. 4C). We also showed overlapping regions with significant differences in flexibility, cohesion and disjointedness in Fig. 4D. However, no significant difference in the nodal-level promiscuity was found between the WCGs and controls. The detailed information for these brain regions with significant differences is listed in Table 3.

**Table 3:** Significant difference in the nodal-level flexibility, cohesion and disjointedness between the WCGs and non-athlete controls

Index	Index of Power Atlas	ROIs	Network	*t-*value	*P*_(FDR)_
Flexibility	25	PostCG_R	SMN	−4.45	0.022
	33	PostCG_L	SMN	−4.31	0.022
	51	MCC_L	CON	−4.514	0.027
	79	MTG_L	DMN	−4.448	0.022
	86	AG_L	DMN	−3.833	0.030
	227	Putamen_L	SUN	−4.300	0.022
Cohesion	25	PostCG_R	SMN	−3.980	0.045
	33	PostCG_L	SMN	−3.739	0.049
	51	MCC_L	CON	−3.918	0.045
	70	ROL_L	AUN	−3.496	0.049
	79	MTG_L	DMN	−4.181	0.045
	86	AG_L	DMN	−4.162	0.045
	160	Lingual_L	VSN	−3.401	0.049
	227	Putamen_L	SUN	−4.187	0.045
	258	SPG_R	DAN	−4.000	0.045
	259	IPG_L	DAN	−3.458	0.045
Disjointedness	25	PostCG_R	SMN	−5.488	0.022
	51	MCC_L	CON	−4.970	0.027
	79	MTG_L	DMN	−3.888	0.030

A positive (negative) *t*-value indicates that the dynamic network parameter was significantly higher (lower) in the WCGs than in the controls. SMN, sensorimotor network; CON, cingulo-opercular network; AUN, auditory network; DMN, default mode network; VSN, visual network; FPN, frontoparietal network; SAN, salience network; SUN, subcortical network; VAN, ventral attention network; DAN, dorsal attention network; PostCG, postcentral gyrus; AG, angular gyrus; MCC, middle cingulate cortex; MTG, middle temporal gyrus; SPG, superior parietal gyrus; IPG, inferior parietal gyrus; ROL, Rolandic operculum; L/R, left/right hemisphere.

### Temporal core and periphery organization

Figure 4E and F shows the distribution of the temporal core (10% lowest flexibility values) and periphery nodes (10% highest flexibility values) in the WCGs and controls. We found that the WCGs showed changes in core-periphery organization compared with the controls (Fig. 4G and H). Specifically, in the temporal core, we observed an increase in the SMN (from 5 to 24%) and a decrease in the DMN (from 68 to 59%), FPN (from 9 to 5%) and SAN (from 9 to 5%) from the controls to the WCGs. Regarding the temporal periphery, there was an increase in the DMN (from 9 to 23%) and FPN (from 4 to 13%), alongside a decrease in the DAN (from 14 to 4%) and SUN (from 23 to 14%) from the controls to the WCGs.

### Robustness results

To test the robustness of the result from the WCGs, we repeated the calculation by varying the window size and sliding step in constructing dynamic FC matrices based on the sliding window approach. The results from these additional analyses were consistent with the primary findings, as detailed in Supplementary Tables 1–3. Importantly, the additional analysis confirmed that educational background did not influence the outcomes, as shown in Supplementary Table 4.

## Discussion

Using a multilayer community detection algorithm, we investigated the effect of long-term intensive training on the brain network dynamic reconfiguration in the WCGs. By estimating how brain region changes in the network communities over time, we found lower global-level flexibility, cohesion and disjointedness in the WCGs than in the controls. Additionally, the WCGs showed decreased flexibility and cohesion in the AUN, CON, DMN, DAN, SMN and SUN compared with the controls. Furthermore, the nodal-level analysis revealed lower flexibility, cohesion and disjointedness in the right PostCG, left MCG and left MTG in the WCGs than the controls. Since flexibility has been associated with skill learning and executive functions, this decreased flexibility suggested a neuroplastic response in the athletes’ brains.

### Decreased flexibility in the world class gymnasts

We observed decreased flexibility across the global, network and nodal levels in the WCGs compared with the controls (Figs 2–4). Flexibility refers to the frequency with which a brain region alters its affiliations with different communities. Previous studies found that brain state flexibility accompanies motor skills acquisition, which requires adaptability in existing brain functions.^9,42,43^ Recent studies based on the multilayer network model observed an inverse U-shaped dynamic pattern shift in flexibility during motor skill learning, with an initial increase followed by a decrease as practice progresses.^9,17,25,44^ For example, Bassett *et al*.^9^ reported this flexibility dynamic pattern in a simple motor learning task, where subjects used their non-dominant hand to respond to visually cued sequences. Subsequently, Li *et al*.^25^ collected resting-state fMRI data from 29 young adults before and after 24 weeks of piano training (all subjects were novices) and found increased flexibility in the visual and auditory network after subjects received musical training. However, mastering piano skills is complex and cannot be achieved in a short period, so these subjects did not exhibit a decrease in flexibility, as seen in simpler motor tasks.^25^

The decreased flexibility also provided evidence for the neural efficiency hypothesis,^45,46^ which assumed experts exhibited lower brain activation compared with less experienced individuals when performing the same cognitive tasks.^47^ This reduction does not indicate diminished ability but rather increased efficiency, as experts rely on optimized neural circuits requiring less cognitive effort.^48^ Our previous rs-fMRI studies found that gymnasts showed decreased intra- and inter-network connectivity compared with controls.^1,5^ This functional evidence suggested that long-term training could optimize brain network organization, maintaining efficient neural processing.

The current study applied a multilayer network approach to analyze the rs-fMRI data and revealed that the dynamic network reconfiguration was affected by long-term intensive training. The decreased flexibility in the WCGs suggested a stable and specialized information processing pattern,^35^ reflecting advanced neural adaptations to minimize the need for frequent reconfiguration of functional networks. These changes were also consistent with the principles of experience-dependent neural plasticity,^49,50^ where extensive practice strengthens commonly used pathways and reduces variability by pruning less essential connections. This neuronal specialization consolidated circuits consistently engaged during training, enabling a streamlined and efficient neural architecture tailored to the demands of gymnastics.

We also observed decreased flexibility at the network level, including the DMN, CON, DAN, AUN and SMN (Fig. 3 and Table 2). These networks with decreased flexibility also showed reduced cohesion (Fig. 3 and Table 2), indicating that the decline in node flexibility was not independent but occurred collectively. This suggested that nodes transitioned between communities less frequently and in a more coordinated manner. Previous studies showed that high cohesion in the initial learning phase facilitates the acquisition of new motor sequences, while long-term intensive training stabilizes these connections, reducing the need for frequent reconfiguration.^21,24^ Additionally, we identified several brain regions exhibiting decreased flexibility and cohesion in the WCGs. These regions include the right PostCG, which is associated with somatosensory processing,^51^ the left MCG, which is involved in motor control and cognitive functions,^52^ the left angular gyrus, which is linked to complex language and number processing,^53^ and the left putamen, which is essential to the motor system.^54^

Previous studies have reported that the flexibility of brain regions can vary depending on their function.^22,39^ By examining the temporal core-periphery organization in the WCGs and controls, we found that the WCGs showed changes in the distribution of temporal core nodes, especially within the SMN, which accounted for 23% of the temporal core regions in WCGs, as opposed to only 5% in controls (Fig. 4). Although we observed decreased flexibility across multiple networks (Fig. 3), the most significant change was in the SMN. This change could be attributed to gymnasts’ long-term intensive training, which likely leads to highly specialized and efficient neural pathways in regions directly involved in motor control and coordination. The SMN, which links the sensory input with motor commands,^24^ is perhaps the most essentially related to gymnastics.^1^ The changes in SMN may suggest that the WCGs have automated specific motor patterns, reflecting the proficient execution of specialized movements.^5^

### Limitation

Several limitations need to be acknowledged. First, the sample size of 13 WCGs is relatively small, which may limit the statistical power and the generalizability of the findings. Future studies should expand the subject pool to include athletes from various sporting disciplines to ensure more robust and widely applicable results. Second, the cross-sectional design of this study failed to monitor changes in brain plasticity over time and establish causality. The result of decreased brain flexibility is based on cross-sectional data rather than longitudinal tracking. Future studies should incorporate longitudinal data to capture these temporal changes for understanding the relationship between long-term intensive training and brain adaptation. Third, an additional limitation of our study was the absence of behavioural data. Future studies would be better to include performance metrics, cognitive assessments, or physical fitness evaluations to provide multidimensional information for understanding how long-term intensive training influences brain dynamic properties in athletes. Finally, our study did not fully consider potential genetic and gender influences, which might impact functional activity and related brain organization. It is important to note that while genetics may contribute to athletic performance, the extent of its influence remains uncertain as there is no definitive evidence linking specific genetic factors to athletic ability.^55^

## Conclusion

Using a multilayer community detection approach, we analyzed the dynamic configurations of brain networks in the WCGs. We found a specific neuroplasticity response in these athletes, characterized by decreased flexibility across the global, network and nodal levels. This study advanced our knowledge of how intensive physical training affects brain network flexibility, offering valuable insights into the neural basis of motor skill acquisition.

## Supplementary Material

fcaf083_Supplementary_Data

## Data Availability

The data supporting the current study are available from the corresponding author upon reasonable request. The code for the multilayer modularity algorithm can be accessed at https://github.com/GenLouvain/GenLouvain. Flexibility, promiscuity, cohesion and disjointedness were calculated using the Network Community Toolbox, available at https://commdetect.weebly.com. Brain visualizations were performed using Nilearn, accessible at https://github.com/nilearn.
