# Supplementary material for: Dynamic reconfiguration of brain functional networks in world class gymnasts: a resting-state functional MRI study
Source: Brain Commun. 2025 Feb 19;7(2):fcaf083. doi: 10.1093/braincomms/fcaf083 (PMC11891512; doi:10.1093/braincomms/fcaf083)
Supplement: fcaf083_Supplementary_Data [file fcaf083_supplementary_data.docx]

**Supplementary materials**

**Title Page**

**Dynamic reconfiguration of brain functional networks in world class gymnasts: a resting-state functional MRI study**

Bolin Cao^1^, Yu Guo^1^, Fengguang Xia^2^, Lunxiong Li^3^, Zhanbing Ren^4^, Min Lu^5^,Jun Wang^6^, Ruiwang Huang^1🖂^

^1^ School of Psychology; Center for Studies of Psychological Application; Guangdong Key Laboratory of Mental Health and Cognitive Science, Key Laboratory of Brain, Cognition and Education Sciences, Ministry of Education; South China Normal University, Guangzhou 510631, China.

^2^ Institute for Brain Research and Rehabilitation, South China Normal University, Guangzhou 510631, China

^3^ Key Laboratory of Brain, Cognition and Education Science, Ministry of Education, China; Institute for Brain Research and Rehabilitation, and Guangdong Key Laboratory of Mental Health and Cognitive Science, South China Normal University, 510631 Guangzhou, China

^4^ Department of Physical Education, Shenzhen University, Shenzhen 518060, China

^5^ Institute of Psychology, Chinese Academy of Sciences (Key Laboratory of Mental Health, Chinese Academy of Sciences)/Department of Psychology, University of Chinese Academy of Sciences, Beijing 100101, China

^6^ Faculty of Psychology, State Key Laboratory of Cognitive Neuroscience and Learning and IDG/McGovern Institute for Brain Research, Beijing Normal University, Beijing, China

^🖂^ Correspondence should be addressed to

Ruiwang Huang, Ph.D.

School of Psychology

South China Normal University

Guangzhou 510631, China

Tel/Fax: +86 (0)20-8521 6033

Email: ruiwang.huang@gmail.com

**Supplementary Table 1** Significant difference in global- and network-level flexibility, cohesion, and disjointedness between the world class gymnasts (WCGs) and non-athlete controls (windows width = 20 TRs and step = 1 TR)

| Indices | | Flexibility | | | Promiscuity | | | Cohesion | | | | Disjointedness | | | |
| --- | --- | --- | --- | --- | --- | --- | --- | --- | --- | --- | --- | --- | --- | --- | --- |
|  |  | *t-*value | | *p*_(FDR_*_)_* | *t-*value | *p*_(FDR)_ | | *t-*value | | *p*_(FDR)_ | | *t-*value | | *p*_(FDR)_ | |
| Global | | -3.633 | 0.001 | | -0.921 | | 0.366 | | -3.552 | | 0.001 | | -2.938 | | 0.007 |
| Network | AUN | -3.528 | 0.012 | | -1.141 | | 0.506 | | -3.397 | | 0.013 | | -2.891 | | 0.042 |
|  | CON | -3.210 | 0.013 | | -0.959 | | 0.506 | | -3.032 | | 0.018 | | -3.791 | | 0.010 |
|  | DMN | -2.584 | 0.028 | | -0.952 | | 0.506 | | -2.572 | | 0.025 | | -1.939 | | 0.218 |
|  | DAN | -3.414 | 0.012 | | -0.850 | | 0.506 | | -3.482 | | 0.013 | | -0.802 | | 0.660 |
|  | FPN | -2.814 | 0.020 | | 0.864 | | 0.506 | | -2.969 | | 0.018 | | -0.541 | | 0.660 |
|  | SAN | -1.643 | 0.127 | | 1.193 | | 0.506 | | -1.710 | | 0.113 | | -0.609 | | 0.660 |
|  | SMN | -2.849 | 0.020 | | -1.326 | | 0.506 | | -2.877 | | 0.018 | | -1.658 | | 0.223 |
|  | SUN | -2.372 | 0.034 | | 0.740 | | 0.519 | | -2.322 | | 0.037 | | -1.682 | | 0.223 |
|  | VAN | -1.573 | 0.130 | | -0.253 | | 0.803 | | -1.633 | | 0.117 | | 0.018 | | 0.986 |
|  | VSN | -2.505 | 0.029 | | -1.447 | | 0.506 | | -2.618 | | 0.025 | | -0.696 | | 0.660 |

A positive (negative) *t*-value indicates that the dynamic graph parameter was significantly higher (lower) in the WCGs than in the controls. Abbreviation: SMN, sensorimotor network; CON, cingulo-opercular network; AUN, auditory network; DMN, default mode network; VSN, visual network; FPN, frontoparietal network; SAN, salience network; SUN, subcortical network; VAN, ventral attention network; DAN, dorsal attention network.

**Supplementary Table 2** Significant difference in global- and network-level flexibility, cohesion, and disjointedness between the world class gymnasts (WCGs) and non-athlete controls (windows width = 40 TRs and step = 1 TR)

| Indices | | Flexibility | | | Promiscuity | | | Cohesion | | | | Disjointedness | | | |
| --- | --- | --- | --- | --- | --- | --- | --- | --- | --- | --- | --- | --- | --- | --- | --- |
|  |  | *t-*value | | *p*_(FDR)_ | *t-*value | *p*_(FDR)_ | | *t-*value | | *p*_(FDR)_ | | *t-*value | | *p*_(FDR)_ | |
| Global | | -2.668 | 0.014 | | -0.808 | | 0.427 | | -2.670 | | 0.014 | | -1.643 | | 0.114 |
| Network | AUN | -2.866 | 0.036 | | -1.143 | | 0.764 | | -2.790 | | 0.036 | | -1.307 | | 0.363 |
|  | CON | -2.314 | 0.076 | | -0.712 | | 0.764 | | -2.179 | | 0.068 | | -2.463 | | 0.221 |
|  | DMN | -2.174 | 0.082 | | -0.516 | | 0.764 | | -2.171 | | 0.068 | | -1.557 | | 0.363 |
|  | DAN | -2.572 | 0.047 | | -0.518 | | 0.764 | | -2.844 | | 0.036 | | -0.305 | | 0.763 |
|  | FPN | -0.947 | 0.354 | | 0.674 | | 0.764 | | -1.386 | | 0.219 | | 1.438 | | 0.363 |
|  | SAN | -0.961 | 0.354 | | 0.746 | | 0.764 | | -0.987 | | 0.335 | | -0.648 | | 0.582 |
|  | SMN | -2.773 | 0.036 | | -2.297 | | 0.315 | | -2.781 | | 0.036 | | -1.860 | | 0.363 |
|  | SUN | -1.676 | 0.154 | | 0.220 | | 0.920 | | -1.549 | | 0.194 | | -1.171 | | 0.363 |
|  | VAN | -0.985 | 0.354 | | 0.090 | | 0.929 | | -1.331 | | 0.219 | | 1.257 | | 0.363 |
|  | VSN | -2.056 | 0.087 | | -0.595 | | 0.764 | | -2.213 | | 0.068 | | -0.747 | | 0.578 |

A positive (negative) *t*-value indicates that the dynamic graph parameter was significantly higher (lower) in the WCGs than in the controls. Abbreviation: SMN, sensorimotor network; CON, cingulo-opercular network; AUN, auditory network; DMN, default mode network; VSN, visual network; FPN, frontoparietal network; SAN, salience network; SUN, subcortical network; VAN, ventral attention network; DAN, dorsal attention network.

**Supplementary Table 3** Significant difference in global- and network-level flexibility, cohesion, and disjointedness between the world class gymnasts (WCGs) and non-athlete controls (windows width = 30 TRs and step = 2 TRs)

| Indices | | Flexibility | | | Promiscuity | | | Cohesion | | | | Disjointedness | | | |
| --- | --- | --- | --- | --- | --- | --- | --- | --- | --- | --- | --- | --- | --- | --- | --- |
|  |  | *t-*value | | *p*_(FDR)_ | *t-*value | *p*_(FDR)_ | | *t-*value | | *p*_(FDR)_ | | *t-*value | | *p*_(FDR)_ | |
| Global | | -3.476 | 0.002 | | -0.885 | | 0.385 | | -3.473 | | 0.002 | | 1.713 | | 0.100 |
| Network | AUN | -3.144 | 0.024 | | -1.920 | | 0.680 | | -3.115 | | 0.024 | | -1.662 | | 0.422 |
|  | CON | -2.863 | 0.026 | | -0.929 | | 0.788 | | -2.842 | | 0.024 | | -2.182 | | 0.401 |
|  | DMN | -2.504 | 0.034 | | -0.399 | | 0.867 | | -2.506 | | 0.034 | | -1.588 | | 0.422 |
|  | DAN | -3.684 | 0.013 | | -0.730 | | 0.788 | | -4.203 | | 0.004 | | 0.232 | | 0.858 |
|  | FPN | -1.180 | 0.278 | | 0.450 | | 0.867 | | -1.368 | | 0.206 | | 0.182 | | 0.858 |
|  | SAN | -1.635 | 0.145 | | 0.002 | | 0.998 | | -1.643 | | 0.143 | | -1.069 | | 0.569 |
|  | SMN | -2.548 | 0.034 | | -1.260 | | 0.788 | | -2.641 | | 0.030 | | -0.972 | | 0.569 |
|  | SUN | -2.800 | 0.026 | | -0.760 | | 0.788 | | -2.865 | | 0.024 | | -0.797 | | 0.589 |
|  | VAN | -0.956 | 0.350 | | -0.117 | | 0.998 | | -1.108 | | 0.280 | | 0.733 | | 0.589 |
|  | VSN | -2.028 | 0.078 | | -0.823 | | 0.788 | | -2.084 | | 0.070 | | -0.989 | | 0.569 |

A positive (negative) *t*-value indicates that the dynamic graph parameter was significantly higher (lower) in the WCGs than in the controls. Abbreviation: SMN, sensorimotor network; CON, cingulo-opercular network; AUN, auditory network; DMN, default mode network; VSN, visual network; FPN, frontoparietal network; SAN, salience network; SUN, subcortical network; VAN, ventral attention network; DAN, dorsal attention network.

**Supplementary Table 4** Significant difference in global- and network-level flexibility, cohesion, and disjointedness between the world class gymnasts (WCGs) and non-athlete controls, adjusted for age, sex, education level, and mean FD as covariates

| Indices | | Flexibility | | | Promiscuity | | | Cohesion | | | | Disjointedness | | | |
| --- | --- | --- | --- | --- | --- | --- | --- | --- | --- | --- | --- | --- | --- | --- | --- |
|  |  | *t-*value | | *p*_(FDR_*_)_* | *t-*value | *p*_(FDR)_ | | *t-*value | | *p*_(FDR)_ | | *t-*value | | *p*_(FDR)_ | |
| Global | | -3.381 | 0.002 | | -0.883 | | 0.386 | | -3.306 | | 0.003 | | -2.522 | | 0.019 |
| Network | AUN | -3.094 | 0.015 | | -1.916 | | 0.691 | | -3.033 | | 0.009 | | -1.797 | | 0.059 |
|  | CON | -2.860 | 0.028 | | -0.957 | | 0.812 | | -2.695 | | 0.032 | | -3.065 | | 0.162 |
|  | DMN | -2.488 | 0.030 | | -0.296 | | 0.843 | | -2.418 | | 0.032 | | -2.292 | | 0.267 |
|  | DAN | -3.651 | 0.030 | | -0.995 | | 0.843 | | -3.856 | | 0.032 | | -0.952 | | 0.267 |
|  | FPN | -1.615 | 0.030 | | 0.283 | | 0.843 | | -1.945 | | 0.032 | | 0.343 | | 0.451 |
|  | SAN | -1.684 | 0.036 | | 0.189 | | 0.852 | | -1.707 | | 0.041 | | -1.107 | | 0.451 |
|  | SMN | -2.713 | 0.056 | | -1.448 | | 0.852 | | -2.761 | | 0.048 | | -1.685 | | 0.451 |
|  | SUN | -2.643 | 0.134 | | -0.298 | | 0.852 | | -2.620 | | 0.082 | | -0.934 | | 0.451 |
|  | VAN | -1.168 | 0.135 | | 0.237 | | 0.852 | | -1.202 | | 0.114 | | -0.064 | | 0.817 |
|  | VSN | -2.200 | 0.256 | | -0.820 | | 0.852 | | -2.275 | | 0.243 | | -1.165 | | 0.950 |

A positive (negative) *t*-value indicates that the dynamic network parameter was significantly higher (lower) in the WCGs than in the controls. Abbreviation: SMN, sensorimotor network; CON, cingulo-opercular network; AUN, auditory network; DMN, default mode network; VSN, visual network; FPN, frontoparietal network; SAN, salience network; SUN, subcortical network; VAN, ventral attention network; DAN, dorsal attention network.
